# Genomic diversity of *Escherichia coli* isolates from backyard chickens and guinea fowl in the Gambia

**DOI:** 10.1099/mgen.0.000484

**Published:** 2020-11-30

**Authors:** Ebenezer Foster-Nyarko, Nabil-Fareed Alikhan, Anuradha Ravi, Nicholas M. Thomson, Sheikh Jarju, Brenda A. Kwambana-Adams, Arss Secka, Justin O’Grady, Martin Antonio, Mark John Pallen

**Affiliations:** ^1^​ Quadram Institute Bioscience, Norwich Research Park, Norwich, Norfolk, UK; ^2^​ Medical Research Council Unit The Gambia at the London School of Hygiene and Tropical Medicine, Atlantic Boulevard Road, Fajara, Gambia; ^3^​ NIHR Global Health Research Unit on Mucosal Pathogens, Division of Infection and Immunity, University College London, London, UK; ^4^​ West Africa Livestock Innovation Centre (WALIC), MB 14, Banjul, Gambia; ^5^​ Warwick Medical School, University of Warwick, Coventry, UK; ^6^​ School of Veterinary Medicine, University of Surrey, Guildford, Surrey, UK

**Keywords:** backyard poultry, chickens, *Escherichia coli*, genomic diversity, guinea fowl, ST155

## Abstract

Chickens and guinea fowl are commonly reared in Gambian homes as affordable sources of protein. Using standard microbiological techniques, we obtained 68 caecal isolates of *Escherichia coli* from 10 chickens and 9 guinea fowl in rural Gambia. After Illumina whole-genome sequencing, 28 sequence types were detected in the isolates (4 of them novel), of which ST155 was the most common (22/68, 32 %). These strains span four of the eight main phylogroups of *E. coli,* with phylogroups B1 and A being most prevalent. Nearly a third of the isolates harboured at least one antimicrobial resistance gene, while most of the ST155 isolates (14/22, 64 %) encoded resistance to ≥3 classes of clinically relevant antibiotics, as well as putative virulence factors, suggesting pathogenic potential in humans. Furthermore, hierarchical clustering revealed that several Gambian poultry strains were closely related to isolates from humans. Although the ST155 lineage is common in poultry from Africa and South America, the Gambian ST155 isolates belong to a unique cgMLST cluster comprising closely related (38–39 alleles differences) isolates from poultry and livestock from sub-Saharan Africa – suggesting that strains can be exchanged between poultry and livestock in this setting. Continued surveillance of *E. coli* and other potential pathogens in rural backyard poultry from sub-Saharan Africa is warranted.

## Data Summary

The genomic assemblies for the isolates reported here are available for download from EnteroBase (http://enterobase.warwick.ac.uk/species/index/ecoli) and the EnteroBase assembly barcodes are provided in File S2 (available in the online version of this article).

Sequences have been deposited in the National Center for Biotechnology Information (NCBI) SRA, under the BioProject ID: PRJNA616250 and accession numbers SAMN14485281 to SAMN14485348 (File S2). Complete assemblies have been deposited in GenBank under the BioProject ID: PRJNA616250 and accession numbers CP053258 and CP053259.

**Impact Statement**

Domestic birds play a crucial role in human society, in particular contributing to food security in low-income countries. Many households in sub-Saharan Africa rear free-range chickens and guinea fowl, which are often left to scavenge for feed in and around the family compound, where they are frequently exposed to humans, other animals and the environment. Such proximity between backyard poultry and humans is likely to facilitate transmission of pathogens such as *Escherichia coli* or antimicrobial resistance between the two host species. Little is known about the population structure of *E. coli* in rural chickens and guinea fowl, although this information is needed to contextualize the potential risks of transmission of bacterial strains between humans and rural backyard poultry. Thus, we sought to investigate the genomic diversity of *E. coli* in backyard poultry from rural Gambia.

## Introduction

The domestic chicken (*Gallus gallus domesticus*) is the most numerous bird on the planet, with an estimated population of over 22.7 billion – 10 times more than any other bird [1]. Since their domestication from the red jungle fowl in Asia between 6000 and 8000 years ago [2, 3], chickens have been found almost everywhere humans live. Other poultry, such as turkeys, guinea fowl, pheasants, duck and geese, are derived from subsequent domestication events across Africa, Europe and the Americas [4]. For example, the helmeted guinea fowl (*Numida meleagris*) originated in West Africa, although domesticated forms of this bird are now found in many parts of the tropics.

Poultry are reared for meat, eggs and feathers [5]. Poultry production is classified into four sectors, based on the marketing of poultry products and the level of biosecurity [6]. Intensive poultry farming falls under sectors 1 to 3, characterized by moderate to high levels of biosecurity, while sector 4 pertains to the ‘backyard’, ‘village’ or ‘family’ poultry system, with few or no biosecurity measures.

Backyard poultry fulfil important social, economic and cultural roles in many societies. Seventy per cent of poultry production in low-income countries comes from backyard poultry [7]. The sale of birds and eggs generates income, while occasional consumption of poultry meat provides a source of protein in the diet. It is estimated that meat and eggs from backyard poultry contribute about 30 % of the total animal protein supply of households in low-income countries [8]. In rural Gambia, backyard poultry can be offered as gifts for newlyweds or sold to solve family needs such as paying school fees, buying new clothes or other household needs [9]. The proximity between backyard poultry and humans may facilitate transmission of pathogens such as *Escherichia coli* between the two host species.

*E. coli* is a generalist bacterium that commonly colonizes the gastrointestinal tract of mammals and avian species [10]. Based on their pathogenic potential, *E. coli* can be divided into three categories: commensals, diarrhoeagenic *E. coli* and extraintestinal pathogenic *E. coli* (ExPEC). ExPEC frequently colonize the gut asymptomatically; however, they possess a wide range of unique virulence factors that enable them to colonize extraintestinal tissues in humans, pets and poultry [11, 12]. A sub-pathotype of ExPEC strains, known as avian pathogenic *E. coli* (APEC), causes colibacillosis – an extraintestinal disease in birds, with manifestations such as septicaemia, air sacculitis and cellulitis [13]. As a result of the high mortality and condemnation of birds associated with avian colibacillosis [14], antimicrobials are often used in intensive farming systems to prevent bacterial infections and treat sick birds – a practice that has been linked to the development of antimicrobial resistance (AMR) in poultry.

Although previous studies have focused on the detection of AMR and documented the emergence of multiple-drug resistance (MDR) in this niche [15–18], little is known about the population structure of *E. coli* in rural backyard poultry. The Gambia does not have genomic data on *E. coli* from poultry prior to this study, and data on the circulating MLST types among poultry *E. coli* strains from sub-Saharan Africa is limited. However, reports from Ghana, Senegal and Nigeria have indicated the prevalence of ST624, ST69, ST540, ST7473, ST155, ST297, ST226, ST10, ST3625 and ST58 among *E. coli* isolates from commercial poultry [19–22]. Given the increased exposure to humans, the natural environment and other animals, the population of *E. coli* in birds raised under the backyard system may differ considerably from those reared in intensive systems. It is also possible that the lineages of *E. coli* within local genotypes of rural poultry might differ between geographical regions. Previous studies have suggested that several *E. coli* clones are shared between poultry and humans, including isolates recovered from clinical cases. These include ST10, ST69, ST95, ST117, ST131, ST155, ST371, ST100, ST88 and ST23, ST38, ST3541, ST3018, ST58, ST6359, ST1011, ST746 and ST2676 [21, 23–29]. The absence of biosecurity measures in backyard poultry farming increases the potential for zoonotic transmission of pathogenic and/or antimicrobial-resistant strains to humans.

In a recent study of commercial broiler chickens, multiple colony sampling revealed that a single broiler chicken could harbour up to nine sequence types of *E. coli* [30]. However, within-host diversity of *E. coli* in backyard poultry, particularly in guinea fowl, has not been well studied and so we do not know how many lineages of *E. coli* can co-colonize a single backyard bird. To address these gaps in our knowledge, we exploited whole-genome sequencing to investigate the genomic diversity and burden of AMR among *E. coli* isolates from backyard chickens and guinea fowl in rural Gambia, West Africa.

## Methods

### Study population

The study population comprised 10 local-breed chickens and 9 guinea fowl from a village in Sibanor in the Western Region of the Gambia (Table 1). Sibanor covers an area of approximately 90 km^2^ and is representative of rural areas in the Gambia [31]. It has a population of about 10, 000. Most of the villagers are subsistence farmers growing peanuts, maize and millet. Households within this community comprise extended family units of up to 15 people, which make up the ‘compound’. All guinea fowl were of the pearl variety, characterized by purplish-grey feathers dotted with white.

**Table 1.** Characteristics of the study population

Sample ID	Poultry species	Gender	Household	Colony picks	Recovered sequence types (No. of colonies per ST)	Phylogroup distribution (STs per phylogroup)
C1	Chicken	Rooster	1	No *E. coli* isolated		
C2	Chicken	Hen	3	1	155 (1)	B1 (155)
C3	Chicken	Rooster	2	5	155 (1), 48 (1), 746 (1) 2461 (1), 542 (1)	A (48, 746, 2461, 542), B1 (155)
C4	Chicken	Rooster	2	5	1423 (1), 337 (1), 9285* (1), 540 (1), 58 (1)	A (540), B1 (1423, 337, 9285*, 58)
C5	Chicken	Hen	2	2	155 (2)	B1 (155)
C6	Chicken	Rooster	2	5	155 (3), 9284* (2)	B1 (155), E (9284*)
C7	Chicken	Rooster	3	5	155 (4), 602 (1)	B1 (155, 602)
C8	Chicken	Rooster	4	5	5286 (1), 2772 (2), 6186 (1), 2165 (1)	A (5286), B1 (2772, 6186, 2165)
C9	Chicken	Hen	5	No *E. coli* isolated		
C10	Chicken	Rooster	5	No *E. coli* isolated		
GF1	Guinea fowl	Rooster	1	5	540 (5)	A (540)
GF2	Guinea fowl	Rooster	1	5	155 (4), 540 (1)	A (540), B1 (155)
GF3	Guinea fowl	Rooster	3	5	540 (2), 443 (1), 6025 (1), 10654* (1)	A (540), B1 (443), D (6025), E (10654)
GF4	Guinea fowl	Rooster	6	5	155(4), 9286* (1)	B1 (155, 9286)
GF5	Guinea fowl	Hen	6	5	155 (2), 4392 (1), 86 (1), 942 (1)	B1 (155, 4392, 86, 942)
GF6	Guinea fowl	Hen	1	5	540 (1), 2067 (4)	A (540), B1 (2067)
GF7	Guinea fowl	Rooster	2	5	212 (4), 155 (1)	B1 (155, 212)
GF8	Guinea fowl	Rooster	7	No *E. coli* isolated		
GF9	Guinea fowl	Rooster	8	5	2614 (2), 295 (1) 196 (1), 2067 (1)	B1 (2614, 295, 196)
Total				68		

*Novel sequence types.

### Sample collection

**Fig. 1.**
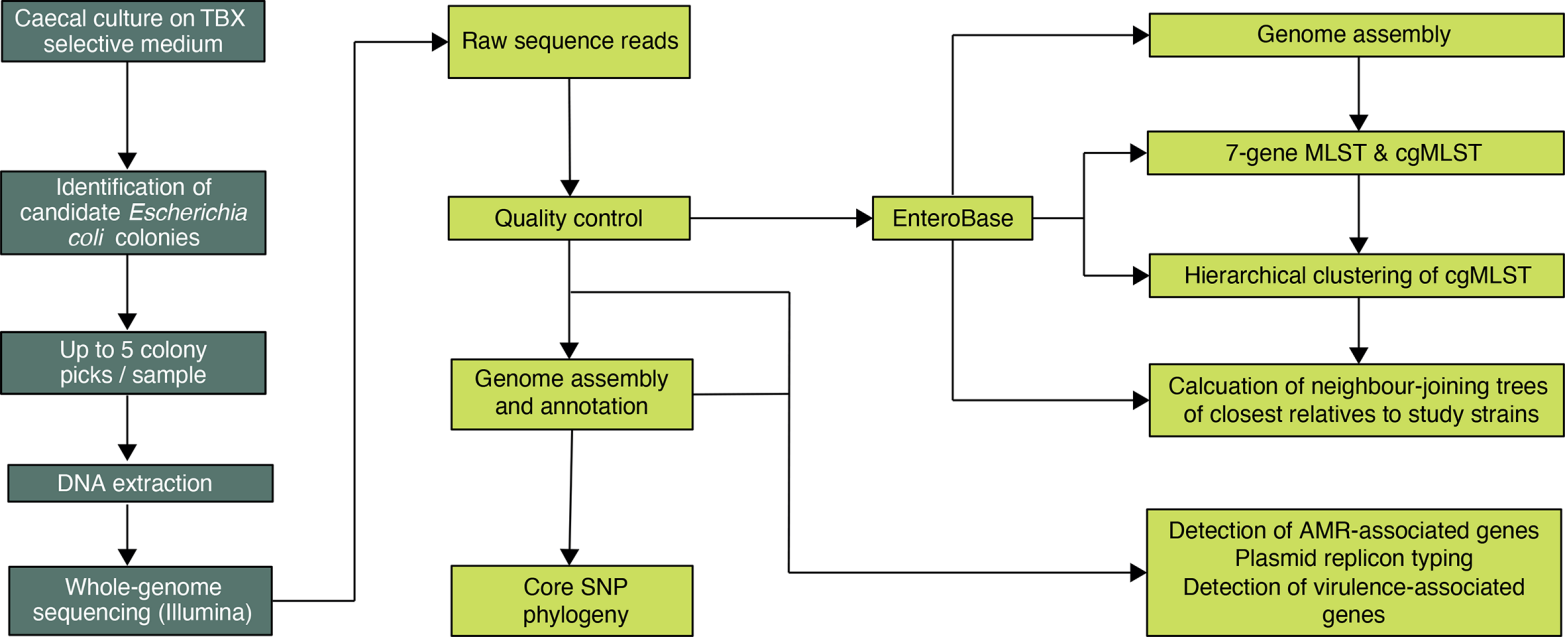
Study sample-processing flow diagram. TBX, tryptone–bile–X-glucoronide agar; MLST, multilocus sequence typing; cgMLST, core genome multilocus sequence typing.

The sampling was done in November 2016. Poultry birds were first observed in motion for the presence of any abnormalities. Healthy-looking birds were procured from eight contiguous households within 0.3–0.4 km of each other and transported to the Abuko Veterinary Station, the Gambia in an air-conditioned vehicle. A qualified veterinarian then euthanized the birds and removed their caeca under aseptic conditions. These were placed into sterile Falcon tubes and flash-frozen on dry ice in a cooler box. The samples were transported to the Medical Research Council Unit The Gambia at the London School of Hygiene and Tropical Medicine labs in Fajara, where the caecal contents were aseptically emptied into new Falcon tubes for storage at −80 °C within 3 h. A peanut-sized aliquot was taken from each sample into a 1.8 ml Nunc tube containing 1 ml of skim-milk-tryptone-glucose-glycerol (STGG) transport and storage medium (Oxoid, Basingstoke, UK), vortexed at 4200 r.p.m. for 2 min and frozen at −80 °C. Fig. 1 summarizes the sample processing flow.

### Microbiological processing

The caecal–STGG suspension was removed from −80^ °^C storage and allowed to thaw briefly on wet ice. A 100 µl aliquot was then taken into 900 µl of physiological saline (0.85 %) and taken through four 10-fold serial dilutions. A 100 µl aliquot each was then taken from the dilutions and uniformly streaked onto tryptone–bile–X-glucoronide agar plates using the spread plate technique. The inoculated plates were incubated at 37 °C for 18–24 h under aerobic conditions. Following overnight incubation, colony counts were determined for raised, translucent and entire colonies that exhibited bluish-green pigmentation typical of *E. coli *. Up to five candidate colonies were selected per sample and sub-cultured on MacConkey agar. These were incubated at 37 °C in air for 18–24 h and stored in 20 % glycerol broth at −80 °C. The isolates from chickens were designated C1–C10, while those from guinea fowl were prefixed by GF1–GF9, followed by the respective colony number (1 up to 5).

### Genomic DNA extraction

Genomic DNA was extracted from overnight broth cultures prepared from each single colony sub-culture using an in-house 96-well plate lysate method as described previously [32]. The DNA was eluted in Tris/Cl (pH, 8.0) and quantified using the Qubit high-sensitivity DNA assay kit (Invitrogen, MA, USA). DNA samples were kept at −20 °C until the Illumina sequencing library preparation. Broth cultures were spun at 3500 r.p.m. for 2 min and lysed using lysozyme, proteinase K, 10 % SDS and RNase A in Tris EDTA buffer (pH 8.0).

### Illumina sequencing

Whole-genome shotgun sequencing of the DNA extracts was performed for all the study isolates on the Illumina NextSeq 500 instrument (Illumina, San Diego, CA, USA) using a modified Illumina Nextera library preparation protocol as described previously [32]. We ran the final pooled library at a concentration of 1.8 pM on a mid-output flow cell (NSQ 500 Mid Output KT v2 300 cycles; Illumina catalogue no. FC-404–2003) according to the manufacturer’s instructions. Following sequencing, FASTQ files were downloaded from BaseSpace to a local server hosted at the Quadram Institute Bioscience.

### Genome assembly and phylogenetic analysis

The raw sequences were initially analysed on the Cloud Infrastructure for Microbial Bioinformatics [33]. This included concatenating paired-end short reads, quality checks with FastQC v0.11.7 [34], trimming of low-quality reads (median quality below a Phred score of~30 and read lengths below 36 bp) and Illumina adapters with Trimmomatic v0.39 [35] and assembly by Spades v3.13.2 [36]. The quality of the assemblies was checked using QUAST v5.0.0, de6973bb [37] and annotation of the draft genomes was carried out using Prokka v1.13.3 [38]. We used the mlst software (https://github.com/tseemann/mlst) to call multilocus sequence types (MLSTs) using the Achtman scheme [39], based on the seven house-keeping genes, *adk*, *fum*C, *gyr*B, *icd*, *mdh*, *pur*A and *rec*A. We used Snippy v4.3.2 (https://github.com/tseemann/snippy) for variant calling and to generate a core-genome alignment, from which a maximum-likelihood phylogenetic tree was reconstructed using RAxML v8.2.4 [40], based on a general time-reversible nucleotide substitution model with 1, 000 bootstrap replicates. We included representative reference genome sequences for the major phylogroups of *E. coli* and *Escherichia fergusonii* as an outgroup (File S1). Given that recombination is widespread in *E. coli* and tends to blur phylogenetic signals [39], we used Gubbins (Genealogies Unbiased By recomBinations In Nucleotide Sequences) [41] to detect and mask recombinant regions of the core-genome alignment prior to the phylogenetic reconstruction. We used the GrapeTree [42] to visualize and annotate phylogenetic trees. We calculated pair-wise single-nucleotide polymorphism (SNP) distances between genomes from the core-genome alignment using snp-dists v0.6 (https://github.com/tseemann/snp-dists).

Subsequently, the short-read sequences were uploaded to EnteroBase [43], an online genome database and integrated software environment that currently hosts more than 138 164 *E. coli* genomes, sourced from all publicly available sequence databases and user uploads. EnteroBase routinely retrieves short-read *E. coli* sequences from the public domain, performs quality control and *de novo* assemblies of Illumina short-read sequences, annotates these and assigns seven-allele MLST (ST) and phylogroups from genome assemblies using standardized pipelines. In addition, EnteroBase assigns unique core-genome MLST (cgMLST) numbers to each genome, based on the typing of 2, 512 genes in *E. coli *.

### Population structure analysis

We utilized the hierarchical clustering (HierCC) algorithm in EnteroBase to assign our poultry genomes to eleven stable clusters designated as HC0 up to HC1100, based on pair-wise differences between genomes at cgMLST alleles. In *Salmonella,* the HC100 or HC200 clusters seem to correspond to long-term strain endemicity, while in *E. coli *, HC1100 corresponds to the seven-allele MLST clonal complexes [43]. The HierCC algorithm therefore lends itself as a very useful tool for the analysis of bacterial population structures at multiple levels of resolution. In a recent study of the population structure of *Clostridioides difficile *, Frentrup *et al*. [44] showed that HierCC allows closely related neighbours to be detected at 89 % consistency between cgMLST pair-wise allelic differences and SNPs. We determined the closest relatives to our study *E. coli* isolates using the HC1100 cluster and reconstructed neighbour-joining trees using NINJA [45]. In order to compare the strain distribution that we observed among our study isolates with what pertains in poultry *E. coli* isolates from elsewhere, we further retrieved genomic assemblies from all publicly available poultry *E. coli* isolates, stratified by their source continent and reconstructed NINJA neighbour-joining trees depicting the prevalence of STs per continent.

### Analysis of accessory gene content

We used ARIBA v2.12.1 [46] to detect virulence factors, antimicrobial resistance genes and plasmid replicons among our study isolates. Briefly, this tool scans the short-read sequences against the core Virulence Factors Database (VFDB) [47] (virulence factors), ResFinder (AMR) [48] and PlasmidFinder (plasmid-associated genes) [49] databases and generates customized outputs, based on a percentage identity of ≥90 % and coverage of ≥70 %. The VFDB-core, ResFinder and PlasmidFinder databases were downloaded on 29 October 2018. As a quality check, the results were confirmed by running ABRicate v0.9.8 (https://github.com/tseemann/abricate) (databases updated 12 October 2020) using the assembled contigs. Virulence factors were visualized by overlaying them onto the phylogenetic tree using the ggtree, ggplot2 and phangorn packages in RStudio v3.5.1.

We determined the prevalence of AMR genes among poultry *E. coli* isolates from the rest of the world, for comparison with what we found in isolates from this study. To do this, we interrogated the downloaded continent-stratified genomes as above using ABRicate v0.9.8 (https://github.com/tseemann/abricate) to predict AMR-associated genes by scanning against the ResFinder database (accessed 28 July 2019), based on a percentage identity threshold of ≥90 % and a coverage of ≥70 %.

### Antimicrobial susceptibility

Due to logistic constraints, a third of the study isolates (20/68, 29 %) were randomly selected for phenotypic susceptibility testing by minimum inhibitory concentrations (MICs). MICs were performed by the agar dilution method [50], according to the European Committee on Antimicrobial Susceptibility Testing v9.0 (EUCAST, 2019) guidelines. Stock solutions of 1000 mg l^−1^ were initially prepared, from which the working solutions were made. For each antibiotic, duplicate twofold serial dilutions (from 32 mg l^−1^ to 0.03 mg l^−1^) were done in molten Müller–Hinton agar (Oxoid, Basingstoke, UK). The results were interpreted according to EUCAST breakpoint tables (http://www.eucast.org). Where EUCAST cut-off values were not available, the recommended cut-off values from the Clinical Laboratory Standards Institute (https://www.clsi.org) were used.

### Oxford Nanopore sequencing

Two novel strains recovered from guinea fowl were long-read sequenced on the Oxford Nanopore platform as follows. Prior to sequencing, DNA fragments were assessed using the Agilent 2200 TapeStation (Agilent catalogue no. 5067–5579) to determine the fragment lengths. Long-read sequencing was carried out using the rapid barcoding kit (Oxford Nanopore catalogue no. SQK-RBK004). Libraries were prepared following the manufacturer’s instructions. An input DNA concentration of 400 ng was used for the library preparation and a final concentration of 75 µl of the prepared library was loaded onto an R9.4 MinION flow cell. The final concentration of the library pool was assessed using the Qubit high-sensitivity DNA assay (Invitrogen, MA, USA).

### Hybrid assembly and analysis of plasmids and phages

The long reads were base-called with Guppy, the Oxford Nanopore Technologies’ post-sequencing processing software (https://nanoporetech.com/). The base-called FASTQ files were then concatenated into a single file each and demultiplexed based on their respective barcodes, using the qcat Python command-line tool v1.1.0 (https://github.com/nanoporetech/qcat). We performed hybrid assemblies of the Illumina and Nanopore reads with Unicycler v0.4.8.0 [51]. The quality of the hybrid assemblies was assessed with QUAST v5.0.0, de6973bb [37]. The hybrid assemblies were then analysed for the presence of plasmids and prophages using ABRicate PlasmidFinder and PHASTER [52] respectively. Annotations of the assemblies were carried out using Prokka v1.13.3 [38].

## Results

### Study population

We analysed 19 caecal samples obtained from 10 chickens and 9 guinea fowl. Fifteen out of the 19 (79 %) samples yielded growth of *E. coli* on culture, from which 68 colonies were recovered (5 colonies from each of 13 birds, 2 from a single bird, and 1 colony from another bird).

### Sequence type and phylogroup distribution

**Fig. 2.**
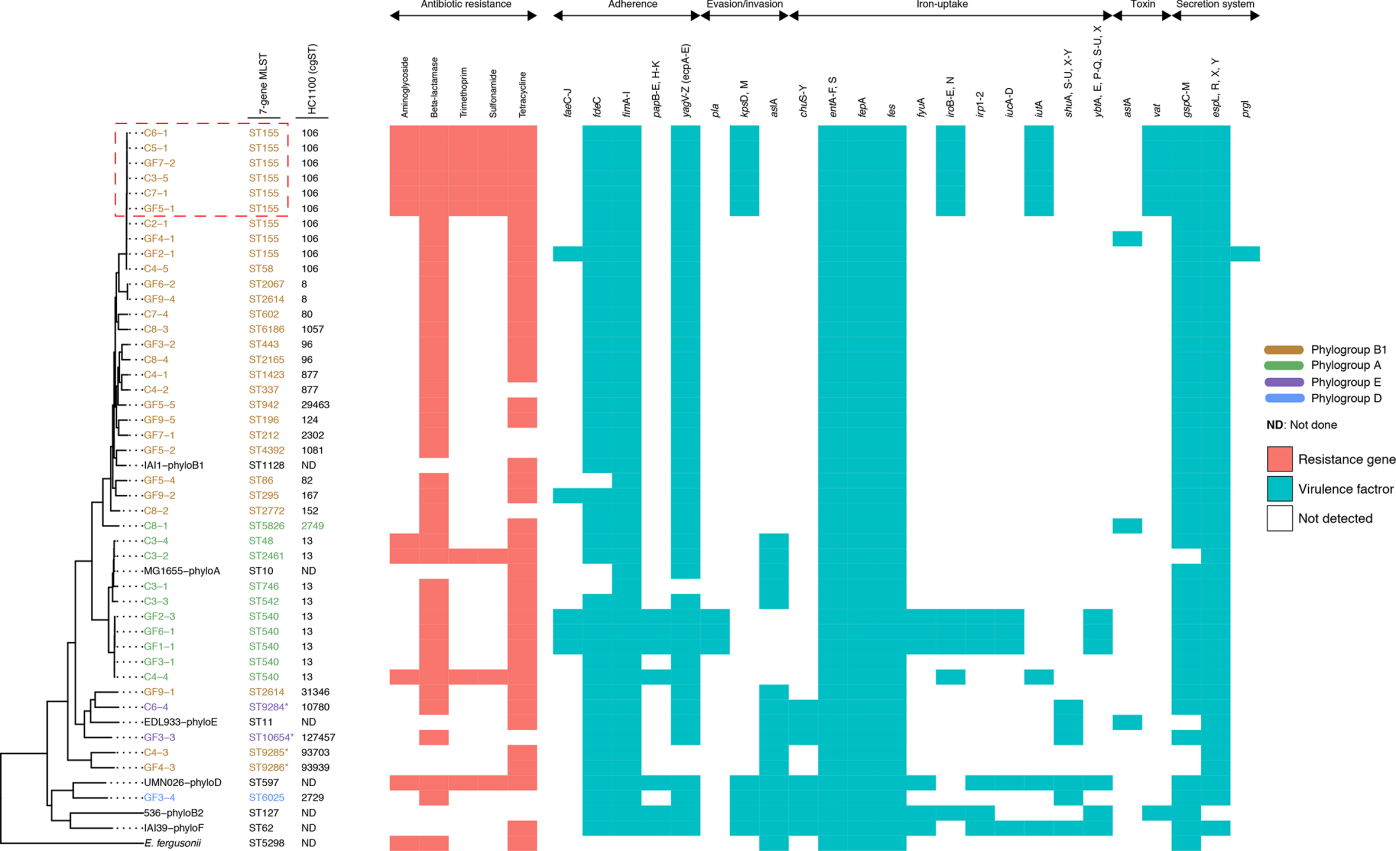
A maximum-likelihood phylogeny of the study isolates reconstructed with RAxML, based on non-repetitive, non-recombinant core SNPs, using a general time-reversible nucleotide substitution model with 1000 bootstrap replicates. The tip labels indicate the sample names, with the respective Achtman sequence types (STs) and HC1100 (cgST complexes) indicated next to the sample names. The colour codes indicate the respective phylogroups to which the isolates belong. The outgroup and the other *E. coli* reference genomes denoting the major *E. coli* phylogroups are in black. Asterisks (*) are used to indicate novel STs. Overlaid on the tree are the predicted antimicrobial resistance genes and virulence factors for each isolate. The virulence genes are grouped according to their function. Chicken isolates are denoted ‘C’ and guinea fowl samples ‘GF’, with the suffix indicating the colony pick. We have not shown multiple colonies of the same Achtman ST recovered from a single individual – in such instances, only one representative isolate is shown. Nor have we shown virulence factors that were detected only in the reference genomes. The red box highlights multi-drug-resistant isolates that concurrently harbour putative fitness and colonization factors that are important for invasion of host tissues and evasion of host immune defences. The full names of virulence factors and their known functions are provided in File S6.

We recovered 28 7-allele sequence types (STs), of which ST155 was the most common (22/68, 32 %). Four of the STs were novel – two from chickens and two from guinea fowl. The allelic profiles of the novel strains are provided in File S2. Seventeen of the 28 STs have previously been isolated from humans or other vertebrates, 6 (ST942, ST2165, ST2461, ST4392, ST5826 and 6186) have not been seen in humans before and 1 (ST6025) only occurred in 1 other isolate in EnteroBase, beside the study strain. However, the source of isolation of this other isolate was not available (Table 2). The isolates were spread over phylogroups B1, A, B2 and D, but most belonged to phylogroups B1 and A, which are home to strains associated with human intestinal infections and avian colibacillosis [53, 54] (Fig. 2). Hierarchical clustering resolved the study strains into 22 cgMLST complexes, indicating a high level of genomic diversity (File S2).

**Table 2.** Prevalence of the study sequence types in EnteroBase

ST	Source	Phylotype	Prevalence in EnteroBase
ST48	Chicken	A	Human, livestock, *Celebes* ape
ST58	Chicken	B1	Human, livestock, poultry
ST86	Guinea fowl	B1	Human, livestock, companion animal, poultry
ST155	Chicken, guinea fowl	B1	Human, poultry, mink, livestock
ST196	Guinea fowl	B1	Human, livestock, companion animal, environment
ST212	Guinea fowl	B1	Human, poultry, deer, companion animal
ST295	Guinea fowl	B1	Human, poultry, livestock, companion animal, environment, food,
ST337	Chicken	B1	Human, rhinoceros, poultry, environment (soil and water)
ST443	Guinea fowl	B1	Human, environment, livestock
ST540	Chicken, guinea fowl	A	Human, environment (water and sewage), livestock, poultry, gull, rabbit, plant, oyster, fish
ST542	Chicken	A	Human, livestock, poultry
ST602	Chicken	B1	Human, poultry, livestock, bird, fish, reptile
ST746	Chicken	A	Human, poultry, fish, livestock, environment (water)
ST942	Guinea fowl	B1	Environment, food, companion animal, livestock
ST1423	Chicken	B1	Human, reptile, livestock
ST2067	Guinea fowl	B1	Human, environment
ST2165	Chicken	B1	Livestock, companion animal, reptile, bird
ST2461	Chicken	A	Sheep, poultry
ST2614	Guinea fowl	B1	Human
ST2772	Chicken	B1	Human, livestock, environment
ST4392	Guinea fowl	B1	Livestock, wild animal, companion animal
ST5826	Chicken	A	Poultry
ST6025	Guinea fowl	D	Unknown source
ST6186	Chicken	B1	Livestock, environment
ST9284	Chicken	E	Novel
ST9285	Chicken	B1	Novel
ST9286	Guinea fowl	B1	Novel
ST10 654	Guinea fowl	D	Novel

*ST6025 occurred in only one other isolate in EnteroBase, beside the study strain. However, the source of isolation of this other isolate was not available.

We generated complete, circular genome assemblies of the two novel sequence types isolated from guinea fowl: ST10654 (GF3-3) and ST9286 (GF4-3). Although neither strain encoded AMR genes or plasmids, GF3-3 contained three prophages (two intact, one incomplete), while GF4-3 harboured four prophages (three intact, one incomplete) (File S3).

### Within-host genomic diversity and transmission of strains

Several birds (12/19, 63 %) were colonized by two or more STs; in most cases, the STs spanned more than two phylotypes (Table 1). In two chickens, all five colony picks belonged to distinct STs. We observed some genetic diversity among multiple colonies of the same ST recovered from the same host (Table 3a). Most of these involved variants that differed by 0–4 SNPs, i.e. variation likely to have arisen due to within-host evolution. However, in one instance, pair-wise SNP differences (ranging from 4 to 255) suggested independent acquisition of distinct clones. Pair-wise SNP analysis also suggested transmission of strains (including MDR isolates) between chickens and between chickens and guinea fowl (Table 3b, c) from the same household (File S4).

**Table 3a.** Within-host single-nucleotide polymorphism diversity between multiple genomes of the same ST recovered from the same bird

Sample ID	Sequence type (ST)	Colonies per ST	Pair-wise SNP distances between multiple colonies of the same ST
C5	ST155	2	0
C6	ST155	3	0
C6	ST9284	2	4
C7	ST155	4	0
C8	ST2772	2	4
GF1	ST540	5	0–3
GF2	ST155	4	0
GF3	ST540	2	2
GF4	ST155	4	0–4
GF5	ST155	2	0
GF6	ST2067	4	0
GF7	ST212	4	4–255
GF9	ST2614	2	0

’C‘ denotes chickens and ‘GF’ denotes guinea fowl.

**Table 3b.** Single-nucleotide polymorphism differences between isolates recovered from chicken 3, chicken 5, chicken 6 and guinea fowl 7. All the isolates in this transmission network encoded resistance to ≥3 classes of antimicrobials

	C3-5	C5-1	C5-2	GF7-2	C6-1	C6-2	C6-3
C3-5	0	0	0	0	0	0	0
C5-1	0	0	0	0	0	0	0
C5-2	0	0	0	0	0	0	0
GF7-2	0	0	0	0	0	0	0
C6-1	0	0	0	0	0	0	0
C6-2	0	0	0	0	0	0	0
C6-3	0	0	0	0	0	0	0

’C’‘ denotes chickens and ‘GF’ denotes guinea fowl.

**Table 3c.** Single-nucleotide diversity differences between isolates recovered from guinea fowls 1, 2 and 6

	GF1-1	GF1-2	GF1-3	GF1-4	GF1-5	GF2-3	GF6-1
GF1-1	0	2	3	1	1	2	3
GF1-2	2	0	3	1	1	2	3
GF1-3	3	3	0	2	2	3	2
GF1-4	1	1	2	0	0	1	2
GF1-5	1	1	2	0	0	1	2
GF2-3	2	2	3	1	1	0	3
GF6-1	3	3	2	2	2	3	0

### Prevalence of AMR, virulence factors and plasmid replicons among the study isolates

**Fig. 3.**
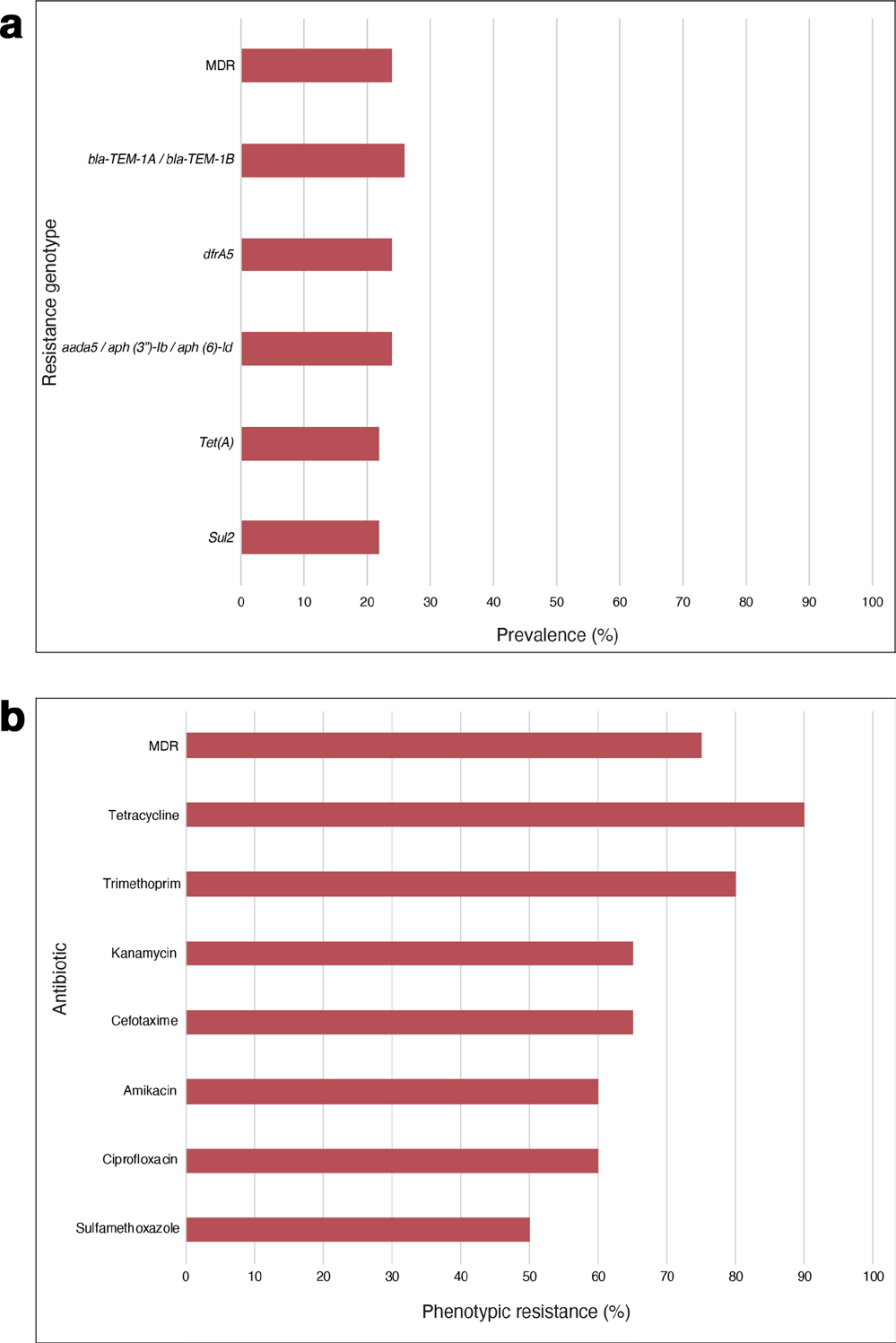
(a) A bar graph showing the prevalence of resistance genes found among the study isolates, using the core Virulence Factors Database (reference 47) (virulence factors), ResFinder (AMR) (reference 48) and PlasmidFinder (plasmid-associated genes) (reference 49) databases, with a cut-off percentage identity of ≥90 % and coverage of ≥70 %. The full list of the resistance genes that were detected is presented in File S5. (b) A bar graph depicting the prevalence of phenotypic antimicrobial resistance in 20 isolates. The results were interpreted using the recommended breakpoint tables from EUCAST (http://www.eucast.org) or the Clinical Laboratory Standards Institute (https://www.clsi.org) where EUCAST cut-off values were not available.

Twenty isolates (20/68, 29 %) harboured at least one AMR gene and 16 (16/68, 24 %) were MDR, i.e. positive for genes predicted to convey resistance to three or more classes of antibiotics (Fig. 3; File S5). Fourteen of the 16 MDR isolates belonged to ST155 – representing 64 % (14/22) of the ST155 isolates recovered in this study. Notable among the resistance genes detected was the class A broad-spectrum beta-lactamase resistance (*bla*_TEM-1A/B_) (18/68, 26 %). Phenotypic resistance was confirmed in >50 % of the isolates tested, with an MDR rate of 75 % (15/20).

Interestingly, the MDR isolates also harboured more genes encoding putative virulence factors than did less-resistant isolates (Fig. 2). Overall, 125 unique virulence-associated genes were detected from the study isolates (File S6). Notably, the virulence and AMR profiles of co-colonizing STs tended to differ from each other.

One or more plasmid replicons were detected in 69 % (47/68) of the study isolates, with 17 plasmid types detected overall (File S7). IncF plasmids were the most common. A single isolate carried the col156 virulence plasmid. The MDR isolates often co-carried large IncF plasmids [IncFIA_1, ~27 kb; IncFIB(AP001918)_1, ~60 kb; IncFIC(FII)_1, ~56 kb]. Scrutiny of annotated assemblies revealed that the resistance genes were often co-located on the same contig as one of the IncF plasmids. In three birds (guinea fowl 2, guinea fowl 5 and guinea fowl 7), co-colonizing strains (belonging to different STs) shared the same plasmid profile. The results of ARIBA ResFinder, PlasmidFinder and VFDB were 100 % concordant with those produced by ABRicate for our study isolates.

### Population dynamics of study strains

**Fig. 4.**
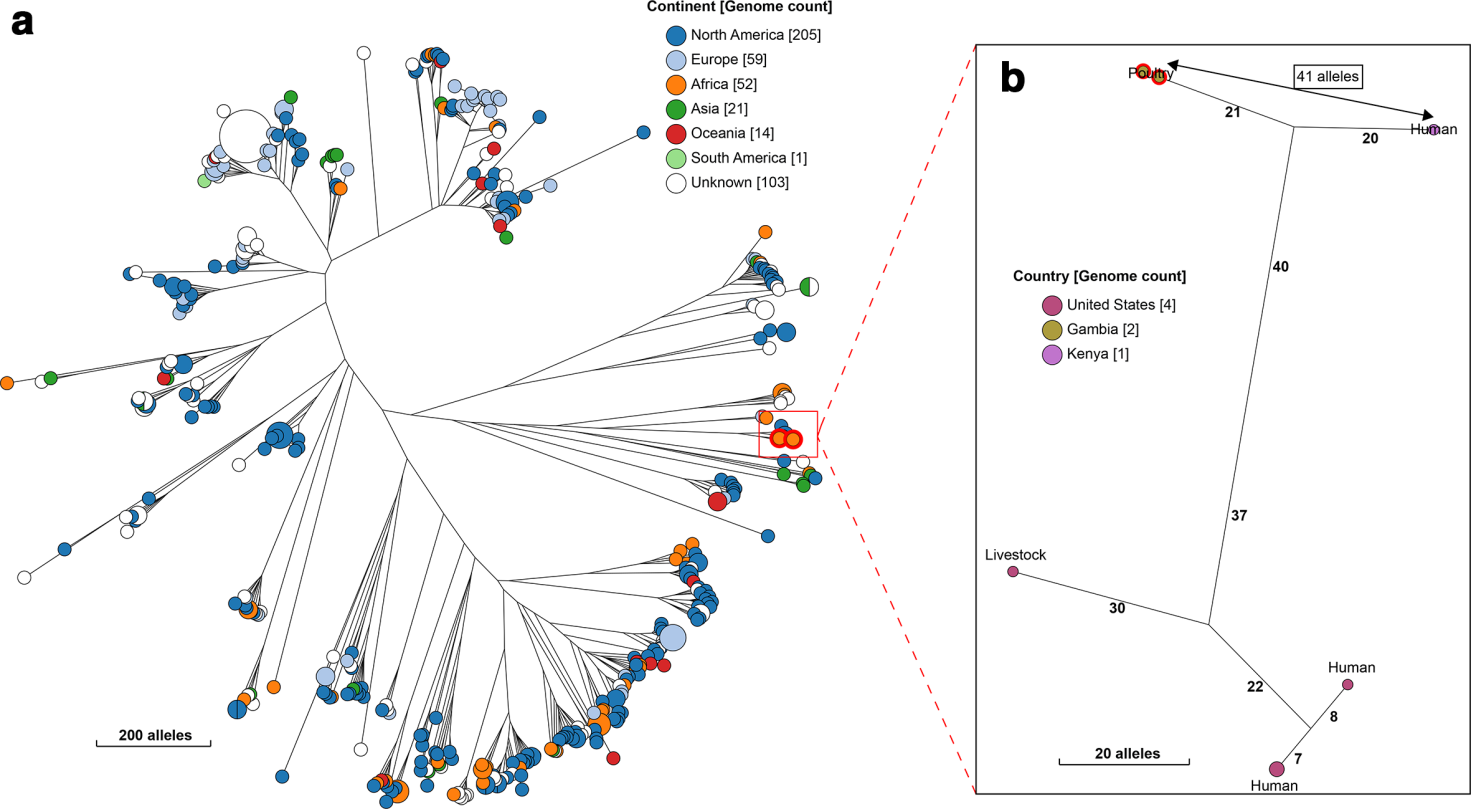
A NINJA neighbour-joining tree showing the phylogenetic relationship between our study ST2772 (Achtman) strain and all other publicly available genomes that fell within the same HC1100 cluster (cgST complex, corresponding to clonal complex in the seven-allele MLST scheme). The locations of the isolates are displayed in the legends, with the genome counts displayed in square brackets. The branch lengths are annotated with the allelic distances separating the genomes. Strains from this study are highlighted in red. The sub-tree (b) shows the closest relatives to the study strains, with the allelic distance separating them displayed with the arrow (41 alleles).

**Fig. 5.**
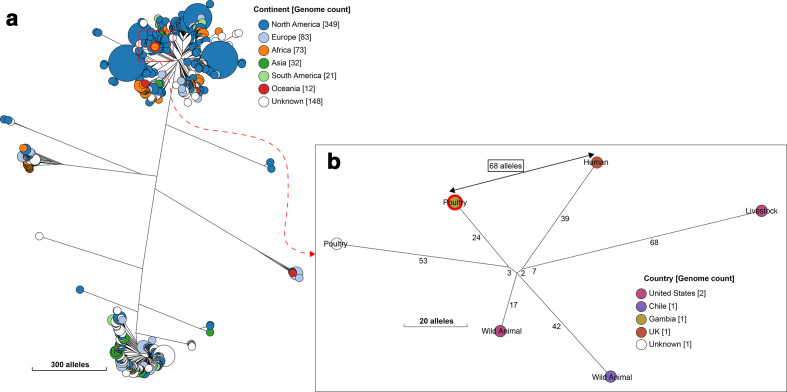
A NINJA neighbour-joining tree showing the phylogenetic relationship between the avian ST4392 (Achtman) strain from this study and all other publicly available genomes that cluster together at HC1100 level (cgST complex, corresponding to clonal complex in the seven-allele MLST scheme). The legend shows the continent of isolation of the isolates, with genome counts displayed in square brackets. Gambian poultry strains are highlighted in red. The study ST strain is separated from a human ST4392 isolate by 68 alleles, as shown in the subtree (b).

Hierarchical clustering analyses provided evidence of genomic relationships between strains from poultry and those from humans (Table 4); however, this warrants further investigation using samples collected from poultry and humans living in close proximity from the same setting. Significant among these were ST2772 and ST4392, which were separated from human isolates belonging to these STs by just 41 and 68 alleles in the core-genome MLST scheme, respectively (Figs 4 and 5). Similarly, ST86, ST6186 and ST602 were closest to isolates from livestock (Figs S9–S11), suggesting possible exchange of strains between livestock species.

**Table 4.** Closest relatives to the Gambian poultry strains

Seven-gene ST	cgST HC100 sub-cluster designation	Study poultry host	Neighbour host	Neighbour’s country of isolation	Allelic distance
ST9286	na	Guinea fowl	Chicken	Gambia (this study)	945
ST9285	na	Chicken	Guinea fowl	Gambia (this study)	945
ST10654	na	Guinea fowl	Unknown avian source	Kenya	1324
ST155	43 137	Chicken and guinea fowl	Poultry	USA	32–34
ST2772	na	Chicken	Human	Kenya	41
ST6186	na	Chicken	Livestock	USA	58
ST540	10 207	Guinea fowl	Human	UK	59
ST58	25 133	Chicken	Unknown	Unknown	59
ST2461	93 699	Chicken	Human	Kenya	64
ST2165	12 281	Chicken	Food	Kenya	66
ST4392	na	Guinea fowl	Human	UK	68
ST602	na	Chicken	Livestock	USA	70
ST540	70 056	Chicken	Food	UK	72
ST540	1320	Guinea fowl	Poultry	USA	73
ST942	na	Guinea fowl	Environment (tap water)	Australia	76
ST212	na	Guinea fowl	Seagull	Australia	81
ST5826	na	Chicken	Water	UK	91
ST1423	27 957	Chicken	Reptile	USA	96
ST337	73 054	Chicken	Reptile	USA	96
ST196	na	Guinea fowl	Human	Kenya	102
ST155	93 719	Chicken	Tanzania	Human	106
ST86	na	Guinea fowl	US	Livestock	131
ST155	73 905	Guinea fowl	Companion animal	USA	137
ST542	93 732	Chicken	Poultry	USA	148
ST746	na	Chicken	Poultry	USA	148
ST295	na	Guinea fowl	Human	Mexico	162
ST48	93 724	Chicken	Unknown	UK	163
ST542	93 697	Chicken	Environment (soil/dust)	USA	194
ST155	73 903	Guinea fowl	Nepal	Human	195
ST443	93 721	Guinea fowl	Unknown	Unknown	224
ST6025	na	Guinea fowl	Unknown	USA	245
ST2614	na	Guinea fowl	Human	PR China	284
ST9284	na	Chicken	Environment (soil/dust)	North America	293
ST2067	na	Guinea fowl	Human	Gambia	458

NA, Not applicable.

By contrast, three of the novel STs from this study (ST10654, ST9285, ST9286) were genetically distinct from anything else in the public domain. These belonged to unique HC1100 clusters in the cgMLST scheme and did not have any relatives in the seven-allele MLST scheme, even after allowing for two mismatches. Two of these (ST10654 from Guinea fowl 3 and ST9286 from Guinea fowl 4) now have complete genomic assemblies.

### The global prevalence of strains and AMR among avian *E. coli* isolates

Phylogenomic analyses of 4, 846 poultry *E. coli* isolates from all over the world revealed that ST155 is common among poultry isolates from Africa and South America (Figs S1 and S2). In contrast, ST117 is prevalent among poultry isolates from Europe and North America (Figs S3 and S4), with ST156 and ST254 being the most common *E. coli* STs found in poultry from Asia and Oceania, respectively (Figs S5 and S6).

Our phylogenetic analyses revealed that ST155 strains from Africa were dispersed among other ST155 isolates from the rest of the world; however, the majority of ST155 strains from this study belonged to a tight genomic cluster, comprising isolates from poultry and livestock from sub-Saharan Africa (separated by 38–39 alleles), except for a single isolate sourced from poultry in the USA. In the cgMLST scheme, all the study ST155 isolates fell into four HC100 sub-clusters (100 alleles difference) (Fig. S7). The largest sub-cluster (sub-cluster 1, HC100_43137) comprised ST155 isolates from this study and isolates from Uganda and Kenya; while sub-clusters 2 (HC100_73903), 3 (HC100_73905) and 4 (HC100_93719) occurred in the Gambia only, although distantly related to isolates from humans and a companion animal (Fig. S8).

Antimicrobial resistance was high across the continents, with the highest prevalence of MDR in South America (100/131, 77 %), followed by Asia (175/249, 70 %) and then Africa (392/591, 66 %) (Table 5; File S8). Of note, the highest percentages of resistance globally were those for broad-spectrum beta-lactamases, while the lowest percentages of resistance were to colistin (File S8). Interestingly, the prevalence of colistin resistance was highest in Europe but did not occur in Oceania and North America.

**Table 5.** Global prevalence of AMR genes

	Europe	Africa	South America	North America	Asia	Oceania
Tetracycline	564/752, 75 %	559/591, 95 %	108/131, 83 %	2480/2975, 83 %	228/249, 92 %	132/148, 90 %
Aminoglycoside	303/752, 40 %	378/591, 64 %	94/131, 72 %	1497/2975, 50 %	172/249, 69 %	56/148, 38 %
Beta-lactamase	303/752, 40 %	246/591, 42 %	127/131, 98 %	933/2975, 31 %	157/249, 63 %	61/148, 41 %
Sulphonamide	338/752, 45 %	377/591, 64 %	84/131, 65 %	1174/2975, 39 %	167/249, 67 %	52/148, 35 %
Trimethoprim	192/752, 25 %	353/591, 52 %	58/131, 45 %	176/2975, 6 %	143/249, 57 %	66/148, 45 %
Chloramphenicol	303/752, 40 %	69/591, 13 %	36/131, 28 %	69/2975, 2 %	131/249, 53 %	0/148, 0 %
Quinolone	51/752, 7 %	144/591, 24 %	24/131, 18 %	17/2975, 1 %	74/249, 30 %	0/148, 0 %
Lincosamide	57/752, 8%	0/591, 0%	12/131, 9 %	0/2975, 0 %	14/249, 6 %	1/148, 1 %
Macrolide	20/752, 3%	79/591, 13%	3/131, 2 %	30/2975, 1%	92/249, 37 %	0/148, 0 %
Fosfomycin	8/752, 1%	4/591, 1%	31/131, 24 %	19/2975, 1%	71/249, 29 %	0/148, 0 %
Streptogrammin	0/752, 0%	0/591, 0%	23/131, 18 %	0/2975, 0 %	0/249, 0 %	0/148, 0 %
Colistin	29/752, 4 %	0/591, 0 %	9/131, 7 %	0/2975, 0 %	119/249, 48 %	0/148, 0 %
MDR	406/752, 54 %	392/591, 66 %	100/131, 77 %	1236/2975, 42 %	175/249, 70 %	56/148, 44 %

The full list of resistance genes that were detected is presented in File S8.

## Discussion

Here, we have described the genomic diversity of *E. coli* from backyard chickens and guinea fowl reared in households in rural Gambia, West Africa. Backyard poultry from this rural setting harbour a remarkably diverse population of *E. coli* strains that encode antimicrobial resistance genes and virulence factors that are important for infections in humans. Furthermore, we provide evidence of sharing of strains (including MDR strains) from poultry to poultry and between poultry, livestock and humans, with potential implications for public health.

Our results reflect the rich diversity that exists within the *E. coli* population from backyard poultry. Although our sample size was small (19 birds), we recovered as many as 28 STs of *E. coli *, 4 of which have not been seen before – even though more than quarter of a million *E. coli* strains had been sequence typed to date (March 2020). Three of our novel STs differed by >945 alleles from their nearest relative. Two of these now have complete assemblies. Also, some of the strains from this study were found in unique cgMLST HierCC clusters containing strains only from this study.

Our results confirm previous reports that phylogroups B1 and A are the dominant phylogroups among *E. coli* isolates from both intensive and backyard poultry [55–58]. Hierarchical clustering analysis suggested that ST155 is common in African poultry. However, most of our ST155 strains belong to a unique cgMLST cluster containing closely related (38–39 alleles differences, and so presumably recently diverged) isolates from poultry and livestock from sub-Saharan Africa, suggesting that strains can be exchanged between livestock and poultry in this setting.

Rural backyard poultry can act as a source of transmission of infections to humans, due to the absence of biosecurity and daily contact with humans [59]. Indirect contact might occur through food or through contact with faeces; for example, by children who are often left to play on the ground [60].

We observed a high prevalence of AMR genes among *E. coli* isolates sourced from African poultry. Similarly, high rates of genotypic MDR were detected among poultry *E. coli* isolates from the rest of the world, with ESBL (various types) being the most significant resistance gene detected. Poultry-associated ESBL genes have also been found among human clinical isolates [61]. Strikingly, most of our ST155 isolates encoded resistance to ≥3 classes of clinically relevant antibiotics, with the highest percentages seen for *bla*_TEM-1_ beta-lactamase and tetracycline (*tetA*). This is worrying, as beta-lactamase-positive isolates are often resistant to several other classes of antibiotics [62, 63].

Our results are consistent with previous studies that reported ST155 isolates to be commonly associated with MDR [64, 65], but differ from other studies that have reported a low prevalence of AMR in backyard poultry. For example, in a study that compared the prevalence of ESBL genes in backyard poultry and commercial flocks from West Bengal, India, none of the 272 *E. coli* isolates from backyard birds harboured any ESBL gene [66], while 30 % of commercial birds carried ESBL genes. The absence of resistance in that study was attributed to a lack of exposure to antimicrobials. Similarly, *E. coli* from organic poultry in Finland were reported to be highly susceptible to most of the antimicrobials studied and no ESBL resistance was detected [67].

Although tetracycline is commonly used in poultry farming for therapeutic purposes [68], resistance to this antibiotic is known to be prevalent in poultry, even in the absence of the administration of this antibiotic [69]. Our results also suggest that IncF plasmids may play a role in the dissemination of AMR in our study population. Conjugation assays are needed to confirm the association of these plasmids with the observed resistance genes and the mobilisability of the plasmids and thus, the potential for exchange among co-colonizing strains in a single host; however, these could not be performed due to coronavirus disease 2019 (Covid-19) restrictions.

Many sub-Saharan countries lack clear guidelines on the administration of antibiotics in agriculture, although an increasing trend in the veterinary use of antimicrobials has been documented [70]. The use of antimicrobials in developing countries is likely to increase because of increasingly intensive farming practices [71]. Europe has banned the use of antimicrobials as growth promoters since 2006 [72] and the use of all essential antimicrobials for prophylaxis in animal production since 2011 [73]. However, AMR may be less well controlled in other parts of the world.

Although APEC strains span several phylogroups (A, B1, B2 and D) and serogroups [54], the majority of APEC strains encode virulence genes associated with intestinal or extra-intestinal disease in humans. These include adhesion factors, toxins, iron acquisition genes and genes associated with serum resistance, such as *fyuA*, *iucD*, *iroN*, *iss*, *irp*2, *hlyF*, *vat*, *kpsM* and *ompT*. Although APEC isolates present different combinations of virulence factors, each retains the capability to cause colibacillosis [13, 74]. We did not detect haemolysin or serum survival genes in our study isolates; however, we recovered some of the known markers of intestinal and extraintestinal virulence in some study isolates, such as the enteroaggregative *E. coli* heat-stable enterotoxin and the vacuolating autotransporter toxin (*vat*, *astA*), invasion and evasion factors (*kpsM*, *kpsD*, *pla*) and adherence factors (*fim* and *pap* genes) that are associated with intestinal and extraintestinal infections in humans. Thus, these strains could cause disease in humans, should they gain access to the appropriate tissues.

Several birds were colonized with two or more STs and at least two phylotypes of *E. coli *. This level of diversity is probably a consequence of the frequent exposure of backyard poultry to the environment, livestock and humans. Co-colonization of single hosts with multiple strains may facilitate the spread of AMR- and virulence-associated genes from resistant strains to other bacteria via both horizontal and vertical gene transfer [75]. A high co-colonization rate of *E. coli* has been described in humans [76, 77] and in non-human primates [32], involving pathogenic strains of *E. coli *. Recently, Li *et al*. reported three to nine sequence types of colistin-resistant *E. coli* to co-exist within a single broiler chicken [30]. Here, we report co-colonization with different lineages of *E. coli* in backyard chickens and guinea fowl. Unsurprisingly, co-colonizing strains often had different AMR and virulence patterns.

An obvious limitation of our study is the small sample size. This study could have also been enhanced by sampling *E. coli* from humans within close proximity to our backyard birds, but we could not perform an analysis of *E. coli* from sympatric humans from our study setting due to logistic reasons and funding limitations of our study. Nonetheless, the inclusion of publicly available sequences strengthens our analysis and inference of the population of *E. coli* in this setting. We also could not perform phenotypic susceptibility testing on all isolates. We acknowledge that a minor percentage of genotypic resistance predictions fail to correspond with phenotypic resistance [78].

Taken together, our results indicate a rich diversity of *E. coli* within backyard poultry from the Gambia, characterized by strains with a high prevalence of AMR and the potential to contribute to infections in humans. This, coupled with the potential for the exchange of strains between poultry and livestock within this setting, might have important implications for human health and warrants continued surveillance.

## Supplementary Data

Supplementary material 1

Supplementary material 2
