# Supplementary material for: Genomic diversity of Escherichia coli isolates from backyard chickens and guinea fowl in the Gambia
Source: Microb Genom. 2020 Nov 30;7(1):mgen000484. doi: 10.1099/mgen.0.000484 (PMC8115903; doi:10.1099/mgen.0.000484)
Supplement: Supplementary material 2 [file mgen-7-484-s002.pdf]

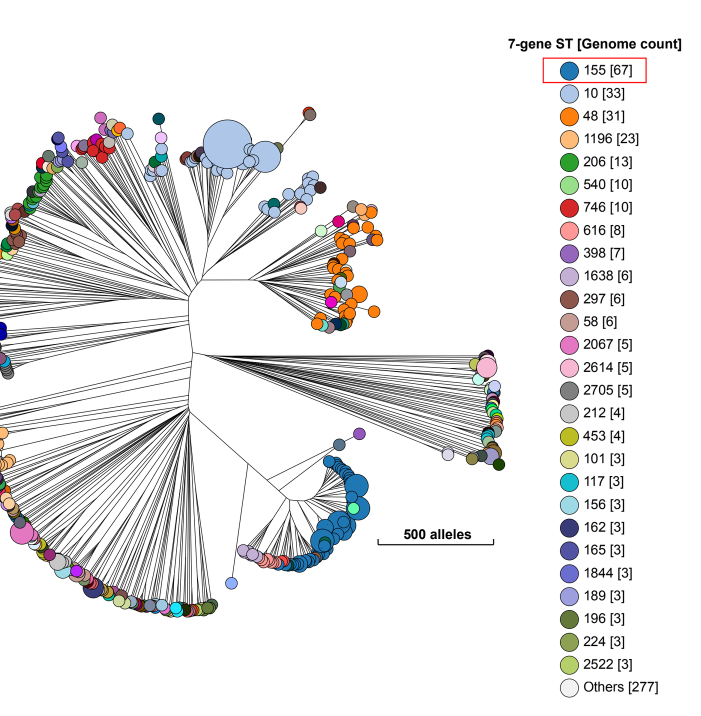

Figure S1. A NINJA neighbour-joining tree of all publicly available *E. coli* poultry isolates from Africa, showing the prevalence of Achtman sequence types (STs). The dominant ST is highlighted with a red box. The legend displays the top 27 STs, with the respective genome counts displayed in square brackets.

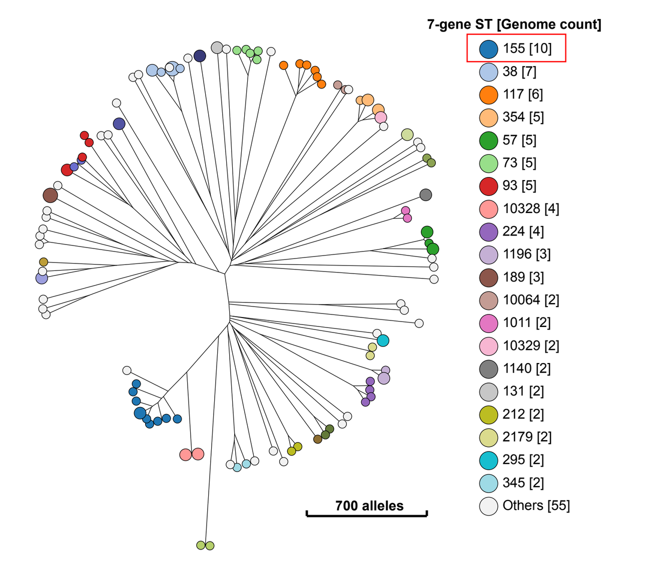

Figure S2. A NINJA neighbour-joining tree of all publicly available *E. coli* poultry isolates from South America, depicting the prevalence of Achtman sequence types (STs). The most common ST found among *E. coli* isolates from this continent is ST155 (highlighted with a red box), similar to Africa (see Figure S1). The top 20 STs are displayed in the legend, with the respective genome counts displayed [in square brackets].

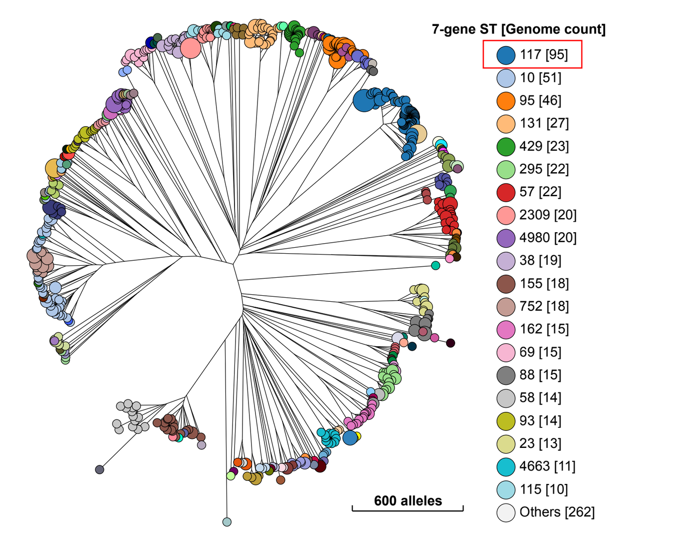

Figure S3. A NINJA neighbour-joining tree of all publicly available *E. coli* poultry isolates from Europe, depicting the prevalence of Achtman sequence types (STs). The top 20 STs are displayed in the legend, with the most common ST among poultry isolates from this continent (ST117) highlighted with a red box. The respective genome count per ST is also displayed.

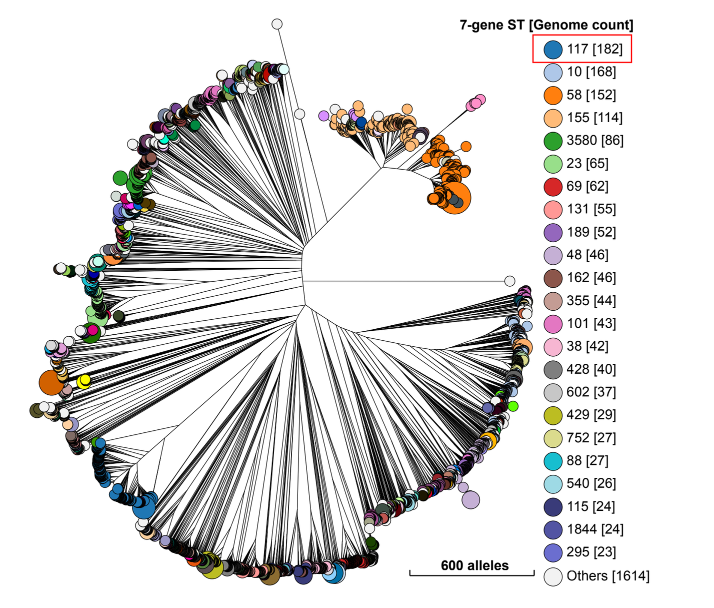

Figure S4. A NINJA neighbour-joining tree of all publicly available *E. coli* poultry isolates from North America, showing the prevalence of Achtman sequence types (STs). The most common ST among poultry isolates from this continent is ST117 (highlighted with a red box). The legend displays the top 23 STs, with the respective genome counts displayed next to the STs.

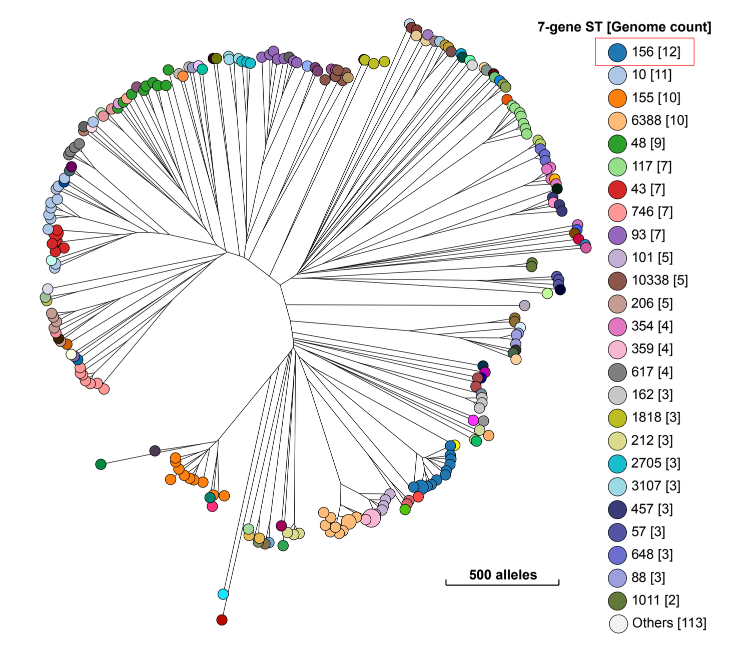

Figure S5. A NINJA neighbour-joining tree of all publicly available *E. coli* poultry isolates from Asia, showing the prevalence of Achtman sequence types (STs). The most common ST among poultry isolates from this continent is ST156 (highlighted with a red box). The legend displays the top 25 STs, with the respective genome counts displayed next to the STs.

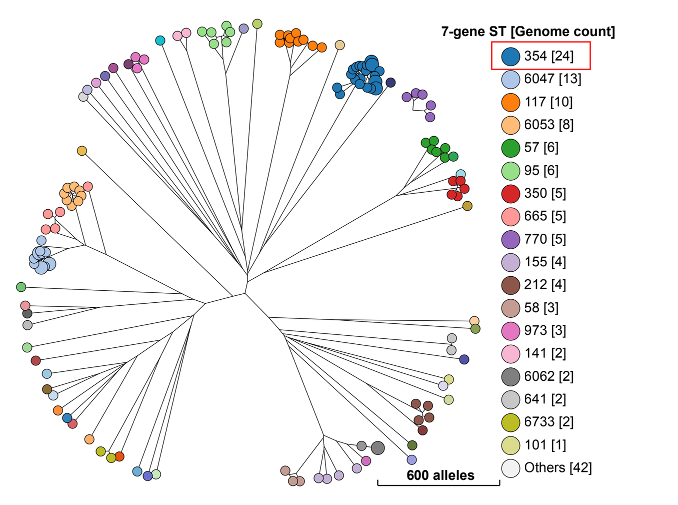

Figure S6. A NINJA neighbour-joining tree of all publicly available *E. coli* poultry isolates from Oceania, depicting the prevalence of Achtman sequence types (STs). The most common ST found among *E. coli* isolates from this continent is ST354 (highlighted with a red box). The first 18 STs are displayed in the legend, with the respective genome counts displayed.

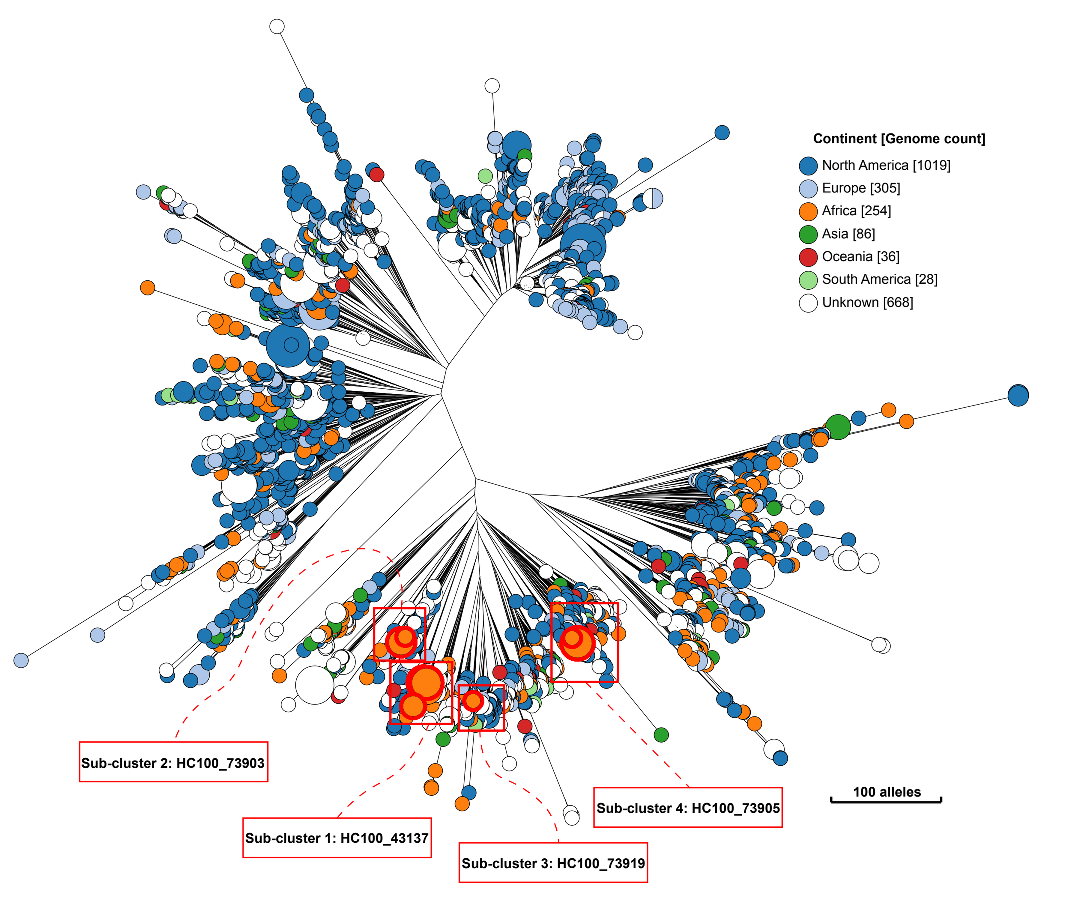

Figure S7. A phylogenetic tree showing the global distribution of *E. coli* ST155 isolates. The study ST155 isolates are highlighted in red. Hierarchical clustering resolved four sub-clusters, encompassing the Gambian ST155 strains, displayed in red boxes. The legend displays the locations of the isolates, with the genome counts depicted in square brackets.

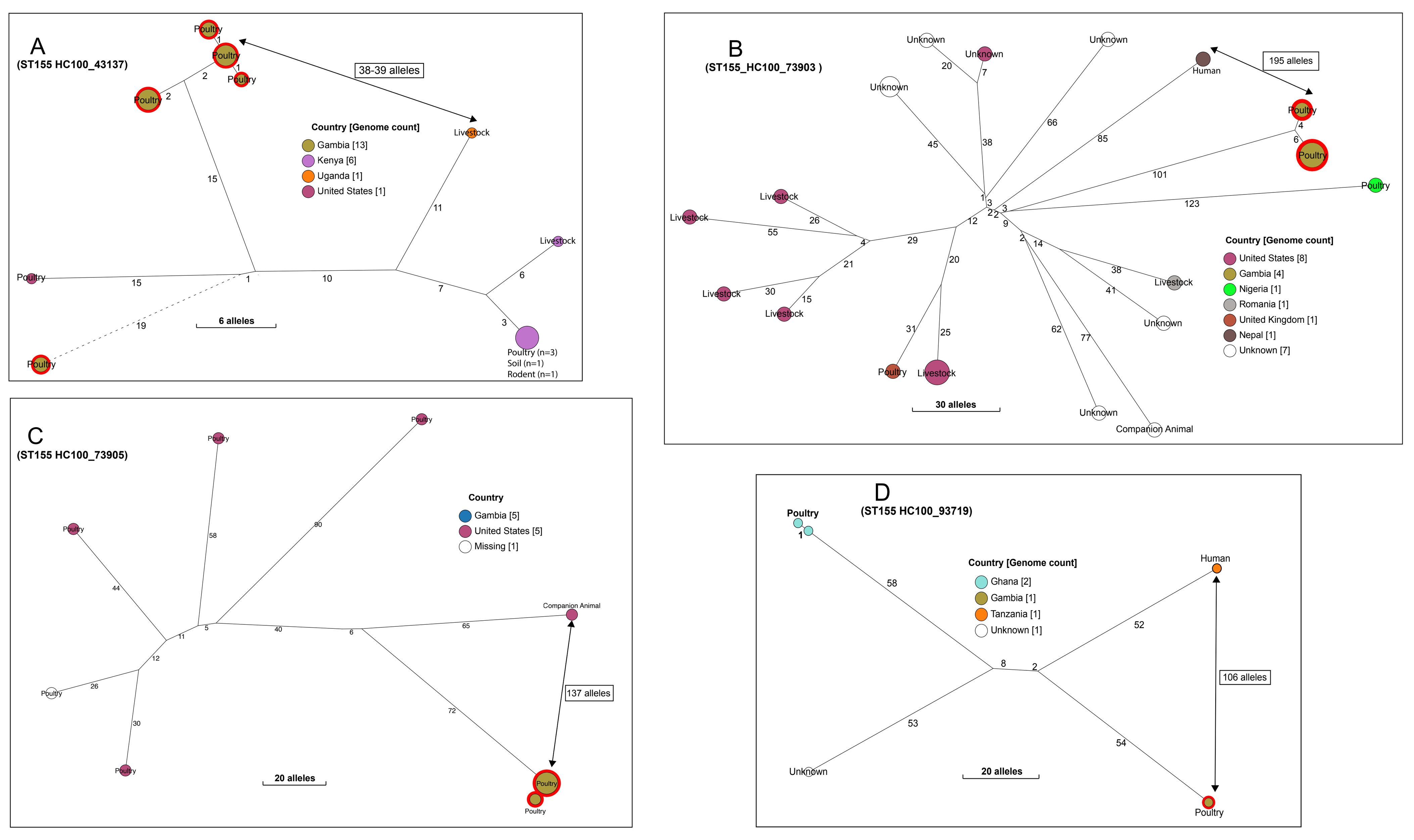

Figure S8. NINJA phylogenetic trees showing the sub-clusters for the study ST155 population within the cgMLST hierarchical clustering scheme. The largest sub-cluster (HC100_43137) (A) encompassed most of the study ST155 isolates (13/22, 59%), which were closely related to isolates from poultry and livestock in sub-Saharan Africa (separated by 38-39 alleles). Sub-clusters 2 (HC100_73903) (B), 3 (HC100_73905) (C) and 4 (HC100_93719) (D) were unique to the Gambia, although distantly related to isolates from humans and a companion animal. The red highlights indicate the study ST155 isolates. The locations of the isolates are displayed in the respective legends, with the genome counts indicated in square brackets.

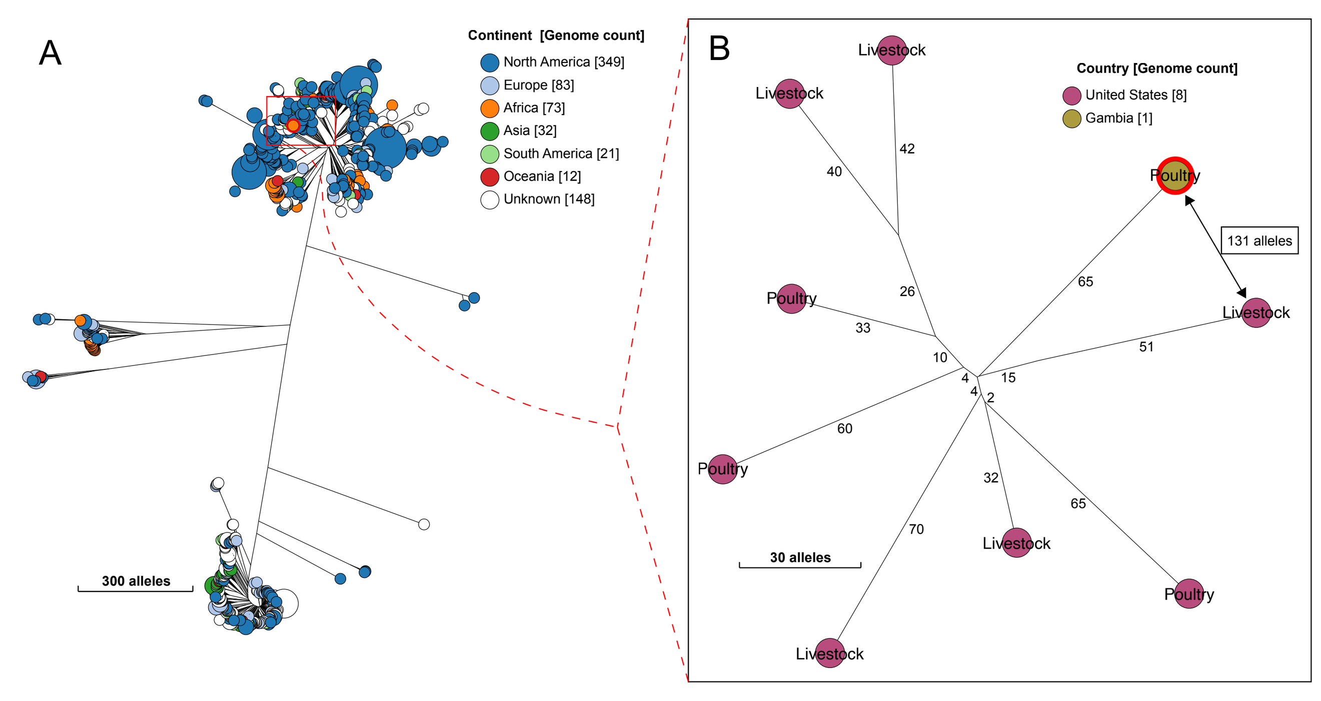

Figure S9. NINJA phylogenetic trees showing the closest neighbours to avian ST86 isolates from this study. The nearest relatives occurred in livestock, depicted with the arrow. The legend indicates the location of isolation, with the genome count displayed in square brackets.

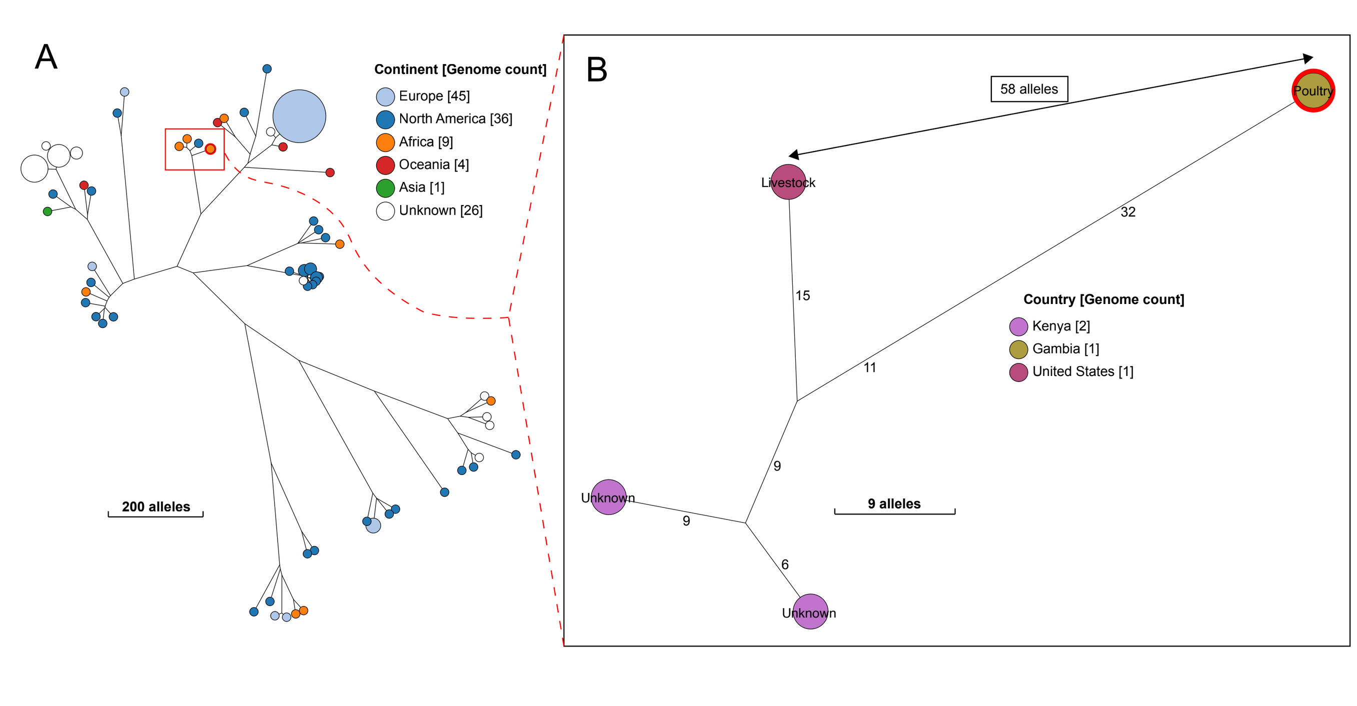

Figure S10. A NINJA phylogenetic tree showing the closest neighbours to avian ST6186 isolates from this study (A). The nearest relatives occurred in livestock from Kenya (B), separated by 58 alleles (depicted with the arrow). The branch lengths display the allelic distance between the genomes. The legend indicates the location of isolation, with the genome count displayed in square brackets.

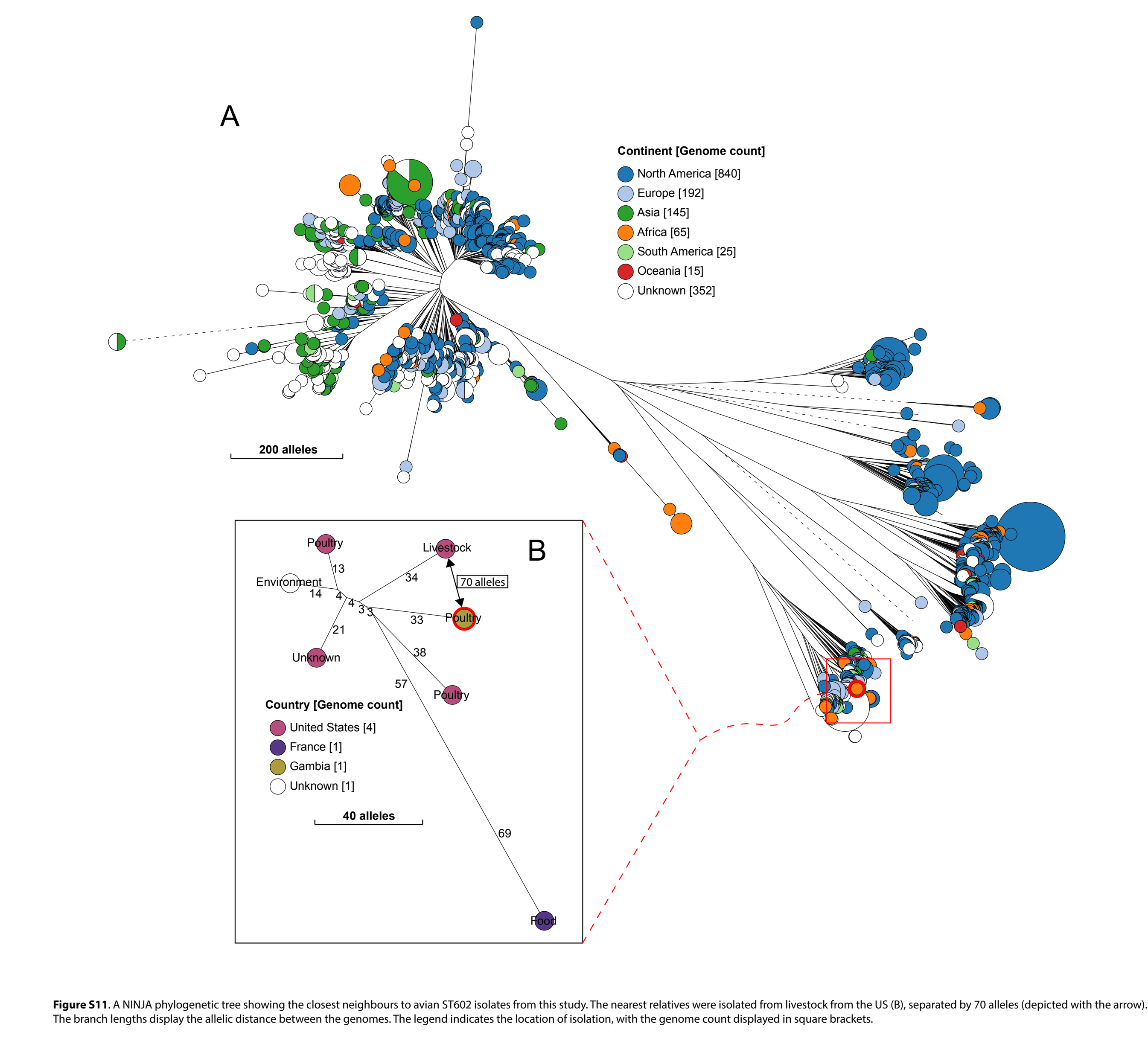

Figure S11. A NINJA phylogenetic tree showing the closest neighbours to avian ST602 isolates from this study. The nearest relatives were isolated from livestock from the US (B), separated by 70 alleles (depicted with the arrow). The branch lengths display the allelic distance between the genomes. The legend indicates the location of isolation, with the genome count displayed in square brackets.
